# Bacterial and viral identification and differentiation by amplicon sequencing on the MinION nanopore sequencer

**DOI:** 10.1186/s13742-015-0051-z

**Published:** 2015-03-26

**Authors:** Andy Kilianski, Jamie L Haas, Elizabeth J Corriveau, Alvin T Liem, Kristen L Willis, Dana R Kadavy, C Nicole Rosenzweig, Samuel S Minot

**Affiliations:** 1 Edgewood Chemical Biological Center, 5183 Black Hawk Rd Bldg E3150 Rm 324, Aberdeen Proving Ground, MD 21010 USA; 2 Defense Threat Reduction Agency, 8725 John J Kingman Rd Stop 6201, Ft. Belvoir, VA 22060-6201 USA; 3 Signature Science, LLC, 8329 N. MoPac Expressway, Austin, TX 78759 USA

**Keywords:** MinION™, Oxford Nanopore, NGS, Nanopore, Amplicon, Pathogen detection, Poxvirus, Escherichia coli, Whole genome sequencing, Long-read sequencing

## Abstract

**Background:**

The MinION™ nanopore sequencer was recently released to a community of alpha-testers for evaluation using a variety of sequencing applications. Recent reports have tested the ability of the MinION™ to act as a whole genome sequencer and have demonstrated that nanopore sequencing has tremendous potential utility. However, the current nanopore technology still has limitations with respect to error-rate, and this is problematic when attempting to assemble whole genomes without secondary rounds of sequencing to correct errors. In this study, we tested the ability of the MinION™ nanopore sequencer to accurately identify and differentiate bacterial and viral samples via directed sequencing of characteristic genes shared broadly across a target clade.

**Results:**

Using a 6 hour sequencing run time, sufficient data were generated to identify an *E. coli* sample down to the species level from 16S rDNA amplicons. Three poxviruses (cowpox, vaccinia-MVA, and vaccinia-Lister) were identified and differentiated down to the strain level, despite over 98% identity between the vaccinia strains. The ability to differentiate strains by amplicon sequencing on the MinION™ was accomplished despite an observed per-base error rate of approximately 30%.

**Conclusions:**

While nanopore sequencing, using the MinION™ platform from Oxford Nanopore in particular, continues to mature into a commercially available technology, practical uses are sought for the current versions of the technology. This study offers evidence of the utility of amplicon sequencing by demonstrating that the current versions of MinION™ technology can accurately identify and differentiate both viral and bacterial species present within biological samples via amplicon sequencing.

**Electronic supplementary material:**

The online version of this article (doi:10.1186/s13742-015-0051-z) contains supplementary material, which is available to authorized users.

## Background

Nanopore-based genomic sequencing has been a concept in development since the first observations of protein nanopores as a means of differentiating nucleotides on a single strand of DNA [1-3]. Protein and solid-state nanopores have been presented as a potential way to determine the sequence of both nucleic acid and protein, and there have been multiple demonstrations of both in the last decade [4-6]. The recent release of the MinION™ by Oxford Nanopore Technologies to a group of alpha-testers makes it the first nanopore-based genomic sequencer to be available for independent assessment. The MinION™ utilizes a customized protein nanopore and combines the sequencing flow cell and electronics into a palm-sized device that is powered and operated via a USB 3.0 connection.

To date, there have been four peer-reviewed publications examining the capabilities of the MinION™ with respect to whole genome sequencing. An initial report by Mikheyev and Tin detailed the performance of the first generation MinION™ reagents and protocols when sequencing purified lambda phage DNA over a 36 hour sequencing run [7]. To demonstrate the utility of the MinION™ for bacterial whole-genome sequencing projects, Quick et al. sequenced *E. coli* K-12 DNA for 72 hours [8]. The MinION™ was also used to decipher the gene organization of a chromosomally-inserted antibacterial resistance cassette in *Salmonella* Typhi [9]. Finally, alignment and SNV software was developed and then utilized to resolve the copy number of a cancer-testis gene family [10]. The first two experiments primarily assessed the per-base accuracy of randomly sheared shotgun sequence reads against a known reference genome, rather than the utility of the MinION™ for *de novo* identification of microbial species and strains. The third study utilized nanopore sequencing with Illumina HiSeq data for error correction. The long reads generated by the MinION™ were instrumental for inferring the gene organization, but only after Illumina sequence data had constructed a scaffold for read mapping. The most recent study utilized only MinION™ reads to resolve human gene copy number while demonstrating the utility of novel alignment approaches for nanopore data.

The main advantages of the MinION™ are its portability and small footprint, easy and quick sample prep, long reads, and flexible run time for data generation. This study targets an application that benefits from these characteristics and determines whether the MinION™ is able to differentiate between closely related viral and bacterial species and strains using amplicon sequencing and 6 hour sequencing runtimes.

## Data description

The raw data collected from these experiments [11] was collected using Oxford Nanopore Technologies’s MinKNOW software (0.46.2.8) as fast5 files containing raw electric signal. These signals were translated into base calls via Oxford Nanopore Technologies’ METRICHOR Agent (r7 2D Basecalling program, version 1.4). The base call data was then transmitted back from the METRICHOR Agent in the form of fast5 files containing sequence read data. The poRe [12] package within R was then utilized to extract the fast5 data into FASTA format for analysis. The raw statistics for the data are depicted in Table 1, and each of the data files exists as a publically accessible fast5 and FASTA file in GigaDB [11]. One raw data set was generated per sample, resulting from 6 hours of MinION™ runtime, and the analytical data is also accessible (as BLASR [13] and LAST [14] generated BAM files) and linked to each raw data set.

**Table 1:** **Raw statistics for bacterial and viral sequencing runs on the MinION™**

				BLASR			LAST		
Dataset	Number of reads	Number of bases	Mean read length	Number of reads aligned	Mean aligned read length	Mean alignment identity	Number of reads aligned	Mean aligned read length	Mean alignment identity
**Lister**	1190	917039	770.6	270	651.4	70.9	751	853.313	66.147
**MVA**	589	492036	835.4	18	353.8	70.1	173	750.873	62.0234
**Cowpox**	1335	1697710	1271.7	40	443.1	69.9	627	758.222	61.5688
**E. coli**	296	847593	2963.5	27	622.9	70.8	47	1143.21	64.8436

## Analyses

MinION™ sequencing libraries were prepared (see Methods) from PCR amplicons generated from cultures of *E. coli* as well as from three poxviruses of varying relatedness (cowpox and vaccinia strains MVA and Lister). From the four datasets that were produced, numbers of reads ranged from 296 – 1,335 with a mean read length of 770 bp - 2,863 bp (Table 1). These reads were generated on R7.0 flow-cell chemistry (Oxford Nanopore) and QC analysis prior to the runs determined between 50–100 active channels per sequencing run. A minority of the data output by the MinION™ aligned well to the known reference sequence using BLASR [13], ranging from 18 – 270 reads aligning with a mean alignment length of 353 bp - 651 bp. Using LAST [14] as the read aligner generated more aligning reads, 47–751, with a greater read length average, 750-1143 bp. The average identity of each alignment ranged from 69.9% – 70.8% using BLASR, with little variation between datasets (Table 1) to 61.6-66.1% with LAST. The origin of the non-aligning reads output by the MinION™ is not known. Most do not align to the CS control DNA and are unidentifiable by BLAST search, with only a small handful aligning to potentially contaminating references. The availability of the datasets [11] will aid in the identification of unaligned reads as the analysis tools for nanopore sequencing continue to be developed.

**Figure 1:**
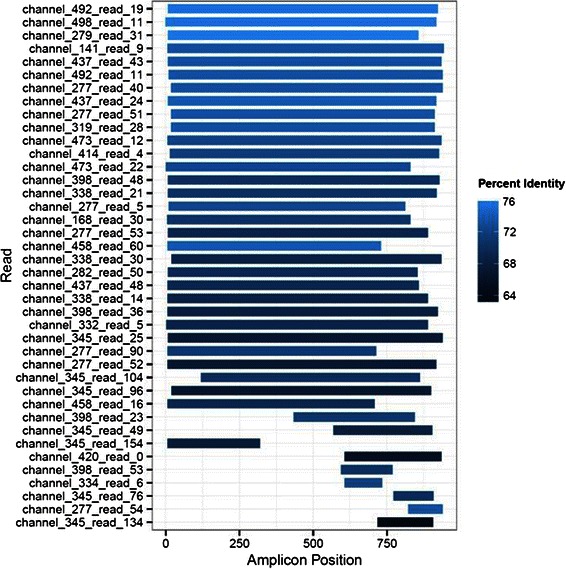
**Distribution of read alignments along the reference using BLASR.** Each mapped read (only 40 reads are depicted here) is depicted by a blue bar. Percent identity to the reference is displayed by the tint, with lighter blue being more identical to the reference. A majority of the reads mapped here were approximately 70-75% identical to the reference. Many mapped reads span the entire length of the reference, represented on the x-axis, with no overhang.

The distribution of read alignments using BLASR across the known reference sequence is shown in Figure 1, with a large proportion of reads completely spanning the amplicon. The majority of reads that align to the known reference sequence do so along the entirety of the read, with little sequence overhanging either end (Figure 1, Additional file 1: Figure S1A). The MinION™ reads generally represent the complete length of the amplicons that were applied to the nanopore sensor, and this is true not only in the vaccinia-Lister dataset but also in the datasets that generated fewer reads mapping to the amplicon (Table 1). The same trends were apparent using LAST to align the data (Table 1, Additional file 1: Figure S1B).

**Figure 2:**
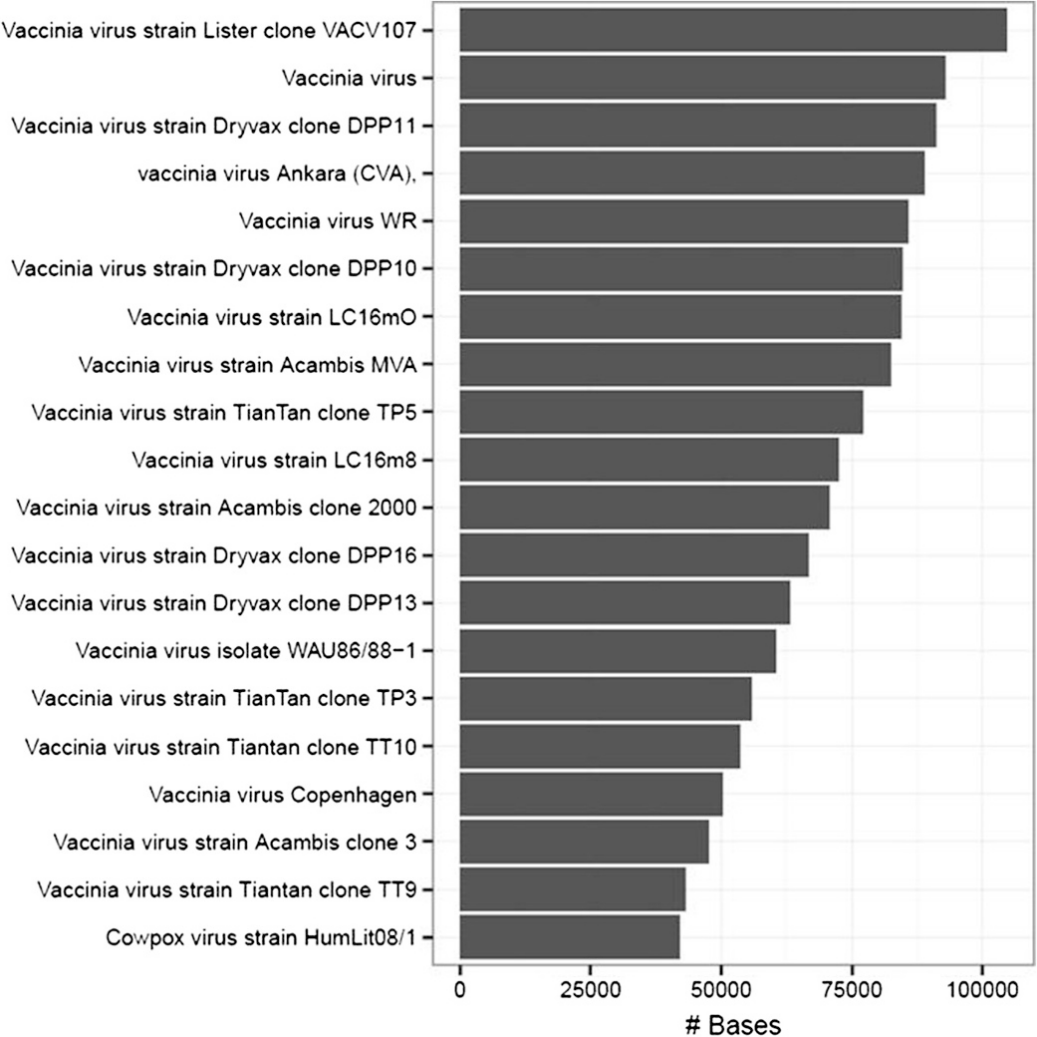
**Bases aligned for vaccinia-Lister sample sequencing run using BLASR.** The number of aligned bases for the vaccinia-Lister dataset are represented by the bars, having been aligned to a reference database containing the complete collection of known targets of the PCR primers that were used.

The reference database used for this comparison was constructed from the known set of similar genes that could potentially be amplified using these primers, included in the GigaDB data [11]. The bacterial reference database included all bacterial 16S rDNA while the viral reference database included all poxvirus sequences that are potentially amplified using the consensus primer pair. During the read mapping process, all potential alignments were retained, so that there was no algorithm used to pick the most likely source of each individual read. In an attempt to represent this novel dataset in the most unbiased manner, the sum of the lengths of each alignment to a given reference was used as a simple summary statistic for the degree to which the input dataset matched that reference. Surprisingly, not only did the correct species receive the largest number of aligned bases, the correct strain within that species was also identified using that metric (Figure 2, Additional file 1: Figure S2).

When aligned to these large databases of reference potential poxvirus amplicons (using BLASR), the correct strain, vaccinia-Lister, is identified as the poxvirus with the largest number of bases aligned from the query dataset (Figure 2, Table 2). Results for the three other datasets are shown in Additional file 1: Figure S2, and similarly to vaccinia-Lister, all of the samples were correctly identified to the species level (E. coli), and to the strain level (cowpox and vaccinia-MVA). Vaccinia-Lister and vaccinia-MVA are over 98% similar to each other, yet these were correctly differentiated based on the mapping characteristics of the datasets for each sample (Table 2). Surprisingly not only did the correct species receive the largest number of aligned bases, the correct strain within that species was also identified using that metric (Table 2). A parallel analysis using the LAST alignment algorithm yielded a similar but potentially less sensitive set of results, with a smaller dynamic range separating true from false positives and less discrimination at the strain level (Table 2). Also calculated was a weighted alignment score for both BLASR and LAST alignments, taking the number of reads multiplied by mean identity (Additional file 1: Figure S4). This metric combines both the depth of the sequence alignments with the mean identity of the aligned reads. The weighted alignment score generally agrees with strain level ID using the number of mapped bases using BLASR and LAST, with vaccinia-Lister the lone incorrectly identified strain using this metric (Additional file 1: Figure S4B). With this set of four organisms including three poxviruses and *E. coli*, amplicon sequencing data produced by the MinION™ was able to correctly identify the source organism and differentiate between closely related poxvirus strains using a variety of read alignment metrics.

**Table 2:** **BLASR/LAST read mapping statistics to genus and species levels from bacterial and viral reads**

Mapping tool	Dataset	Reads aligning to genus	Bases aligning to genus	Reads aligning to species	Bases aligning to species	Reads aligning to non-genus	Bases aligning to non-genus	Bases aligning to genus/bases aligning to non-genus	Genus	Species	Strain
**BLASR**	**Lister**	291	189590	151	97410	172	96260	1.97	Orthopoxvirus	Vaccinia	Lister
	**MVA**	25	9634	21	8381	12	2951	3.26	Orthopoxvirus	Vaccinia	MVA
	**Cowpox**	56	23036	56	23036	14	2533	9.09	Orthopoxvirus	Cowpox	-
	***E. coli***	19	15485	16	12640	7	2083	7.43	Shigella	coli	-
**LAST**	**Lister**	151	140099	29	26967	308	274216	0.51	Orthopoxvirus	Vaccinia	MVA
	**MVA**	44	37511	43	36507	21	18400	2.04	Orthopoxvirus	Vaccinia	MVA
	**Cowpox**	89	72375	89	72375	50	41077	1.76	Orthopoxvirus	Cowpox	-
	***E. coli***	17	20399	17	20399	32	35661	0.57	Escherichia	coli	-

## Discussion

Amplicon sequencing has been utilized as an approach in microbiome studies, cancer mutation identification, and pathogen detection [15-17]. Having the ability to sequence amplicons in a mobile, small-footprint platform is attractive when collecting and analyzing samples in the field, and these qualities are also desired as sequencing methods move towards clinical applications. These methods, data, and results represent a practical and novel application for the current stage of MinION™ nanopore sequencing technology. These data were generated on R7.0 chemistry flow cells, which resulted in as little as 10% of the channels within the flow cell being operational. This is lower than what has been reported by others [8], but it has been suggested that the quick evolution of the flow-cell development might have led to lower yields from R7.0 flow-cell chemistry. It is also possible that the overnight incubation of libraries (Methods) with the supplied motor protein lowered data yield from the amplicon experiments. Recent preliminary reports within the MinION™ alpha-testing community suggest that the latest versions of flow-cell chemistry, in addition to newer DNA preparation methodology, yield better pore usage and generate more data. As this is the first report of nanopore sequenced amplicons, hopefully this will set a benchmark for the improvement of data yield and utility from these types of samples. We expect that as the MinION™ technology improves, amplicon sequencing will generally become cheaper, faster, and more accurate.

These results confirm the observation by others that the per-base accuracy of MinION™ data (65-80%) is below that of other DNA sequencing methods [7,8]. In order for amplicon sequencing to be useful in the face of this high error rate, the rate at which reads align to the correct species and strain must be far higher than the rate of spurious alignments that would lead to false positives. We found that each dataset aligned to the correct reference genome at a 1.95 to 9.04-fold higher rate than the spurious alignments to non-target organisms using BLASR (Table 2), even when using the permissive default settings for the BLASR alignment algorithm [13]. The datasets aligned to the correct reference at a rate of 0.51 to 2.04 using the LAST alignment, but a majority of the top identifications were correct (Table 2). These datasets will serve as a resource to other bioinformatic researchers who are designing pathogen detection algorithms to work with MinION™ data.

Utilizing the option to precisely specify the length of a given sequencing run, sufficient coverage depth to accurately call the amplicon sequence was achieved in 6 hours. The was true for both bacterial and viral species, and the amplicon data generated for cowpox, vaccinia-MVA, and vaccinia-Lister was able to be used to correctly identify these closely related strains. With read-level accuracy at 65-80%, generating significant coverage depth is necessary for extracting accurate consensus sequences. When analyzing a complex mixed sample that might contain an agent of interest such as *E. coli* (5-6mb) or poxviruses (150-280 kb) with a majority of DNA from host or other organisms present, it is likely that the amount of coverage for the agent of interest would be extremely limited after only 6 hours of runtime thus making it difficult to identify or characterize any pathogens within the sample. More accurate identification and characterization can occur using the MinION™ by concentrating the data collection over a particular genomic region via targeted amplification when using nanopore sequencing to determine the biological agent milieu of a complex sample.

While the field of taxonomic classification and pathogen detection within complex mixed DNA samples has developed a set of highly sophisticated algorithms and tools (e.g. [18-21], and many others), none of these have yet been modified or validated for use with nanopore sequence data. Therefore, in this study we have chosen to focus on the rate of alignment of a set of reads against a small reference dataset as a proxy for the broader utility of nanopore data for pathogen detection. The importance of finely tuned and validated detection algorithms is shown clearly by the subtly different results returned by two algorithms, BLASR and LAST, which both attempt to find the optimal set of correct alignments of the same set of reads against the same reference database. The difference between those two aligners barely begins to scratch the surface of the vast parameter space that must be explored to find the best method for translating raw nanopore data into actionable information on the presence of specific organisms within a complex environmental or clinical sample. However, these results indicate that the input nanopore sequence data contains sufficient information in order to drive that detection in a reliable and accurate manner.

To date, the MinION™ work being reported has demonstrated the enormous potential of nanopore sequencing, while also highlighting that for whole genome sequencing approaches improvements will need to be made as the technology matures. By utilizing amplicon sequencing as a method for bacterial and viral detection and differentiation, accurate depictions of what agents are in a sample can be generated. Importantly, this is a practical use for the technology as it exists now, and the methods described here will only become more accurate as nanopore sequencing technology evolves. The methods and results presented here and deposited in GigaDB [11] will help inform and guide the community as applications are sought for the current generation as well as for future iterations of nanopore sequencing technology.

## Methods

Cowpox, Vaccinia-MVA, and Vaccinia-Lister were grown in Vero (Modified Vaccinia Ankara or MVA) or BHK cells (cowpox, Lister) until the development of CPE (cytopathogenic effect). Cells were then harvested and DNA extracted using the DNeasy Blood & Tissue Kit (Qiagen, Valencia, CA, USA). Poxvirus amplicons were then generated by using the consensus primers EACP1 (ATGACACGATTGCCAATAC) and EACP2 (CTAGACTTTGTTTTCTG) [22,23] and HotStarTaq PCR kit (Qiagen). *E. coli* K12 (Affymetrix, Santa Clara, CA, USA) DNA was amplified using the 16S-specific primers adapted from probeBase [24] S-D-Bact-0008-c-S-20-ONT (GCCATCAGATTGTGTTTGTTAGTCGCTAGRGTTYGATYMTGGCTCAG) and S-D-Bact-1391-a-A-17-ONT (GCTTACGGTTCACTACTCACGACGATGGACGGGCGGTGWGTRCA). PCR products were verified via gel electrophoresis then cleaned up with Agencourt AMPure beads (Beckman Coulter, Miami, FL, USA). The amplicons were prepped using genomic preparation kits (SQK-MAP-002) and the methodology from Oxford Nanopore, which is briefly described below and performed as detailed by the nanopore literature from Oxford Nanopore. 1ug of purified amplicon DNA with 50 ng added lambda phage control DNA was end repaired (NEBNext End Repair Module, New England Biolabs, Beverly, MA, USA) and dA-tailed (NEBNext dA-Tailing Module, New England Biolabs). A hairpin adapter was then ligated to the end repaired and dA-tailed amplicons, followed by an overnight incubation with a motor protein (to facilitate entry of the hairpin and complementary DNA strand into the pore). 6ul of the resulting prepared library was diluted in 140ul of MinION™ loading buffer with 4ul MinION™ fuel mix (Oxford Nanopore Technologies, Oxford, UK). The MinION™ flow cells were primed with 300ul MinION™ loading buffer followed by the loading of the sequencing mix. The flow cell was run for 6 hours for each sequencing run.

Template reads in FASTA format were extracted using poRe [12] from the HDF5 files output by the MinION™ base-calling software. Those reads were aligned against a reference dataset containing the set of microbial genomic regions predicted to be amplified by the primers used for each dataset using BLASR [13] with default settings, or LAST [14] using the settings described previously for MinION™ data: match score (r) of 1, gap opening penalty (a) of 1, and gap extension penalty (b) of 1 [8]. Alignment summaries were output by BLASR and LAST and ‘pileup’ files were generated using Samtools [25]. Read percentage identity was calculated as described previously for LAST output [8], and the percentage identity values for BLASR were used as output. R and ggplot2 [26] were used to plot the percent identity and alignment span for each read against its known reference, as well as the number of bases aligned to each reference genome in the larger database. A complete set of instructions for that analysis is provided as supplemental information.

### Availability and requirements

- Project name: Minion viral and bacterial species amplicon sequencing shell scripts
- Project home page: https://github.com/gigascience/paper-kilianski2015
- Operating system: Unix
- Programming language: Bash
- Other requirements: Unix
- License: GPL v3.

## Availability of supporting data

The datasets supporting the results of this article are available in the GigaDB repository [11], and the European Nucleotide Archive under accession number ERP009678 and BioProject PRJEB8656.

## Additional file

Additional file 1: Additional data and scripts. **Figure S1.** Aligned length vs. read length for mapped vaccinia-Lister reads. Dotplot analyzing the aligned length vs. the read length for each mapped read with A) BLASR and B) LAST. **Figure S2.** Aligned bases and top hits for *E. coli*, vaccinia-MVA, and cowpox. Based on the identical workflow for the analysis of the vaccinia-Lister sample, aligned bases and identified top hits are displayed for A) *E. coli*. B) vaccinia-MVA, and C) cowpox. Note that the *Shigella* results for the E. coli dataset are interpreted as true positive results, as the top three 16S hits in panel (C) are >99.8% identical, as measured by pairwise BLASTN. **Figure S3.** Aligned bases and top hits using the LAST aligner. Plots are shown as in Figure S2 for A) *E. coli*, B) vaccinia-Lister, C) vaccinia-MVA, and D) cowpox. **Figure S4.** Weighted alignment scores for BLASR and LAST alignments. Number of total bases multiplied by the mean identity yields weighted BLASR and LAST (respectively) alignments for the A) *E. coli*, B) vaccinia-Lister, C) vaccinia-MVA, and D) cowpox data sets. Supplemental information. Script used in shell and R to align reads and plot the percent identity and alignment span for each read against its known reference, as well as the number of bases aligned to each reference genome in the larger database.

## Abbreviations

BLASR: Basic Local Alignment with Successive Refinement
CPE: Cytopathogenic effect
HDF5: Hierarchical Data Format 5
MVA: Modified Vaccinia Ankara
