# Supplementary material for: Bacterial and viral identification and differentiation by amplicon sequencing on the MinION nanopore sequencer
Source: Gigascience. 2015 Mar 26;4:12. doi: 10.1186/s13742-015-0051-z (PMC4374364; doi:10.1186/s13742-015-0051-z)
Supplement: Additional file 1: Additional data and scripts. — Figure S1. Aligned length vs. read length for mapped vaccinia-Lister reads. Dotplot analyzing the aligned length vs. the read length for each mapped read with A) BLASR and B) LAST. Figure S2. Aligned bases and top hits for E. coli, vaccinia-MVA, and cowpox. Based on the identical workflow for the analysis of the vaccinia-Lister sample, aligned bases and identified top hits are displayed for A) E. coli. B) vaccinia-MVA, and C) cowpox. Note that the Shigella results for the E. coli dataset are interpreted as true positive results, as the top three 16S hits in panel (C) are >99.8% identical, as measured by pairwise BLASTN. Figure S3. Aligned bases and top hits using the LAST aligner. Plots are shown as in Figure S2 for A) E. coli, B) vaccinia-Lister, C) vaccinia-MVA, and D) cowpox. Figure S4. Weighted alignment scores for BLASR and LAST alignments. Number of total bases multiplied by the mean identity yields weighted BLASR and LAST (respectively) alignments for the A) E. coli, B) vaccinia-Lister, C) vaccinia-MVA, and D) cowpox data sets. Supplemental information. Script used in shell and R to align reads and plot the percent identity and alignment span for each read against its known reference, as well as the number of bases aligned to each reference genome in the larger database. [file 13742_2015_51_MOESM1_ESM.doc]

**SUPPLEMENTAL DATA**

A) B)

**Figure S1. Aligned length vs. read length for mapped vaccinia-Lister reads.** Dotplot analyzing the aligned length vs. the read length for each mapped read with A) BLASR and B) LAST.

1. B)

C)

**Figure S2. Aligned bases and top hits for *E. coli*, vaccinia-MVA, and cowpox.** Based on the identical workflow for the analysis of the vaccinia-Lister sample, aligned bases and identified top hits are displayed for A) *E. coli*. B) vaccinia-MVA, and C) cowpox. Note that the *Shigella* results for the *E. coli* dataset are interpreted as true positive results, as the top three 16S hits in panel (C) are >99.8% identical, as measured by pairwise BLASTN.

A) B)

C) D)

**Figure S3. Aligned bases and top hits using the LAST aligner.** Plots are shown as in Figure S2 for A) *E. coli*, B) vaccinia-Lister, C) vaccinia-MVA, and D) cowpox.

1. *E. coli*

1. vaccinia-Lister

1. vaccinia-MVA

1. cowpox

**Figure S4. Weighted alignment scores for BLASR and LAST alignments.** Number of total bases multiplied by the mean identity yields weighted BLASR and LAST (respectively) alignments for the A) *E. coli*, B) vaccinia-Lister, C) vaccinia-MVA, and D) cowpox data sets.

**SUPPLEMENTAL INFORMATION**

Script used in shell and R to align reads and plot the percent identity and alignment span for each read against its known reference, as well as the number of bases aligned to each reference genome in the larger database.

**READ MAPPING**

#!/bin/sh

dataset="$1" #Name to describe dataset

fp=$dataset\_reads.fq #Path to FASTQ file that will be created

fast5_folder="$2" #Folder containing all fast5 files for dataset

ref="$3" #FASTA all possible hits

true_ref="$4" #FASTA true reference

fo=$fp

#Path to executables

blasr='bin/blasr'

samtools='bin/samtools'

pileup_parser='bin/pileup.parser.py'

#poRe must be installed via R -- Version 0.6 used.

if [ ! -s $ref ]; then echo "$ref is empty or does not exist"; exit; fi

if [ ! -s $true_ref ]; then echo "$true_ref is empty or does not exist"; exit; fi

#Extract fastq files from fast5 directory

echo "library(poRe)

extract.run.fastq('$fast5_folder')"| R --no-save --no-restore --vanilla

#Concatenate all of the reads into a single file

cat $fast5_folder/extracted/*template.fastq > $fp

#Make sure the read names don't duplicate

cat $fp |awk '{if(substr($1,1,3)==">ch"){print ">" (NR+1)/2 "_" substr($1,2)}else{print}}' > $fo.temp

mv $fo.temp $fp

#Run BLASR on the whole set and the true ref, outputting per alignment details

$blasr -m 4 $fp $ref > $fo.read.stats

$blasr -m 4 $fp $true_ref > $fo.true.read.stats

$blasr -sam $fp $true_ref | $samtools view -bT $ref - | $samtools sort - $fo.true.srt

$samtools index $fo.true.srt.bam

$samtools mpileup -f $true_ref $fo.true.srt.bam | $pileup_parser > $fo.true.pileup

#Analyze the output data

echo "library(ggplot2)

library(outliers)

pdf(file='$fo.pdf')

#Function to read in FASTQ files

read.fq<-function(fp){

dat<-scan(fp, sep='\n',what='character')

out<-data.frame(name=c(), nucs=c(), qscores=c(), length=c(), stringsAsFactors=FALSE)

while(length(dat)>0){

#If the first line doesn't mark a new sequence, delete the first line and try again

if(substr(dat[1],1,1)!='@'){

dat<-dat[-1]

}else{

title=substr(dat[1],2,nchar(dat[1])) #Name of sequence

dat<-dat[-1]

#How long is the first record?

n<-which(dat=='+')[1]-1

if(length(dat)>=((2*n)+1)){

nucs<-paste(dat[1:n], collapse='')

#Delete the sequence

dat<-dat[(n+2):length(dat)]

qscores<-paste(dat[1:n], collapse='')

out<-rbind(out, data.frame(name=title, nucs=nucs, qscores=qscores, length=nchar(nucs), stringsAsFactors=FALSE))

if(length(dat)>n){

dat<-dat[(n+1):length(dat)]

}else{dat<-c()}

}else{dat<-c()}

}

}

return(out)

}

## ALIGNMENT TO KNOWN TRUE REFERENCE

#What is the true accuracy of each read?

#Read in the stats on the aligned reads

dat<-read.table(file='$fo.true.read.stats', sep=' ', stringsAsFactors=FALSE, header=F)

names(dat)<-strsplit('qName tName score percentSimilarity qStrand qStart qEnd qLength tStrand tStart tEnd tLength mapQV', ' ')[[1]] #BLASR -m 4 format

dat\$readname<-sapply(dat\$qName, function(x){strsplit(x, '/')[[1]][1]})

#Only keep the best alignment for each read

dat<-do.call(rbind, lapply(unique(dat\$readname), function(r){

t.dat<-subset(dat, readname==r)

if(nrow(t.dat)==1){return(t.dat)}

t.dat<-subset(t.dat, score==max(t.dat\$score))

return(t.dat)

}))

dat<-dat[rev(order(dat\$score)),]

dat\$align_len<-abs(dat\$tEnd-dat\$tStart)

print(qplot(data=dat, align_len, percentSimilarity, main='Similarity to Known Reference by Read Length', geom='point',

xlab='Aligned Read Length', ylab='Percent Similarity')+theme_bw())

#Get the lengths of the input reads from the FASTQ

reads<-read.fq('$fp')

#Mark each read as one that aligned or not

reads\$Aligned<-sapply(reads\$name, function(x){x %in% dat\$readname})

#Plot the read lengths according to whether or not they aligned

print(qplot(data=reads, length, geom='histogram', xlab='Read Length', ylab='Number of Reads',

main='Read Length Distribution by Alignment to Reference')+theme_bw()+facet_grid(Aligned~., scales='free_y'))

#For the reads that aligned, plot the read length against the alignment length

print(qplot(data=dat, qLength, align_len, xlab='Read Length', ylab='Alignment Length',

main='Read Length vs. Alignment Length', geom='point')+theme_bw()) #+geom_abline(slope=1, intercept=0))

#Plot the length and quality of each read along the true reference

read.pos<-do.call(rbind, lapply(1:nrow(dat), function(q){

data.frame(Read=dat\$readname[q], Percent_Identity=dat\$percentSimilarity[q], Position=dat\$tStart[q]:dat\$tEnd[q])

}))

#Don't plot more than 40 reads, so that the labels remain visible

max.reads<-40

if(length(unique(read.pos\$Read))>max.reads){read.pos<-subset(read.pos, Read %in% sample(unique(read.pos\$Read), max.reads))}

print(qplot(data=read.pos, Position, Read, geom='line', color=Percent_Identity, size=5, xlab='Amplicon Position')+theme_bw()+guides(size=FALSE)+scale_color_continuous(name='Percent Identity'))

#Show the coverage along the length of the reference

pileup<-read.table(file='$fo.true.pileup', sep='\t', fill=T, stringsAsFactors=FALSE,header=T)

print(qplot(data=pileup, pos, cov, xlab='Position', ylab='Depth', geom='bar', stat='identity', main='Coverage of Reference')+theme_bw())

## ALIGNMENT TO LARGE DATABASE OF REFFERENCES

#Read in the stats on the aligned reads

dat<-read.table(file='$fo.read.stats', sep=' ', stringsAsFactors=FALSE, header=F)

names(dat)<-strsplit('qName tName score percentSimilarity qStrand qStart qEnd qLength tStrand tStart tEnd tLength mapQV', ' ')[[1]] #BLASR -m 4 format

dat\$readname<-sapply(dat\$qName, function(x){strsplit(x, '/')[[1]][1]})

dat\$align_len<-abs(dat\$tEnd-dat\$tStart)

#Calculate the aligned read length X percentSimilarity

dat\$weighted_len<-dat\$align_len*dat\$percentSimilarity

#Reformat the names (because greengenes gave it something much too long)

dat\$tName<-gsub('\"', '', dat\$tName)

dat\$tName<-gsub(\"'\", '', dat\$tName)

dat\$tName<-sapply(dat\$tName, function(x){x<-strsplit(x, '_')[[1]];if(substr(x[2],1,2)=='gi'){x<-x[-2]};return(paste(x, collapse='_'))})

dat\$tName<-sapply(dat\$tName, function(x){y<-strsplit(x, '_')[[1]]; if(length(y)<10){return(x)}; y<-y[7:(length(y)-3)];return(paste(y, collapse=' '))})

dat\$tName<-sapply(dat\$tName, function(x){if(substr(x,1,1) %in% as.character(c(1:10))){y<-strsplit(x, '_')[[1]]; return(paste(y[-1], collapse='_'))};return(x)})

dat\$tName<-gsub('_', ' ', dat\$tName)

#Keep a single alignment for each read against each reference, choosing the alignment with the highest weighted length (align_len * percentSimilarity)

dat<-do.call(rbind, lapply(unique(dat\$readname), function(r){

t.dat<-subset(dat, readname==r, stringsAsFactors=FALSE)

if(nrow(t.dat)==1){return(t.dat)}

return(do.call(rbind, lapply(unique(t.dat\$tName), function(Ref){

t.t.dat<-subset(t.dat, tName==Ref, stringsAsFactors=FALSE)

if(nrow(t.t.dat)==1){return(t.t.dat)}

return(subset(t.t.dat, weighted_len==max(t.t.dat\$weighted_len), stringsAsFactors=FALSE))

})))

}))

#Count the number of aligned reads for each reference

ref<-data.frame(name=unique(dat\$tName), stringsAsFactors=FALSE)

ref\$nreads<-sapply(ref\$name, function(r){sum(dat\$tName==r)})

ref\$alignedBases<-sapply(ref\$name, function(r){sum(dat\$align_len[dat\$tName==r])})

#Score based on aligned bases and identity

ref\$score<-sapply(ref\$name, function(r){sum(dat\$weighted_len[dat\$tName==r])})

#Order the table by that score

ref<-ref[rev(order(ref\$score)),]

ref<-ref[min(nrow(ref), 20):1,]

print(ref)

ref\$name<-factor(ref\$name, levels=ref\$name, ordered=TRUE)

print(qplot(data=ref, nreads, alignedBases, geom='point',

main='Number of Reads and Number of Bases Aligned by Reference',

xlab='Number of Reads Aligned', ylab='Number of Bases Aligned')+theme_bw())

print(qplot(data=ref, name, nreads, geom='bar', stat='identity',

main='Number of Reads Aligned', xlab='', ylab='# Reads')+coord_flip()+theme_bw())

print(qplot(data=ref, name, alignedBases, geom='bar', stat='identity',

main='Number of Bases Aligned', xlab='', ylab='# Bases')+coord_flip()+theme_bw())

print(qplot(data=ref, name, score, geom='bar', stat='identity',

main='Weighted Alignment Score', xlab='', ylab='Score')+coord_flip()+theme_bw())

dev.off(); q()

" | R --no-save --no-restore

**READ COUNTS**

#!/bin/bash

echo "Dataset nReads nBases meanLength alignedReads meanAlignLen meanIden" > read.counts.tsv

for f in *_reads.fq; do

n=${f%_reads.fq}

nReads=$(cat $f | tr '\n' '\t' | sed 's/@channel/\n@channel/g'|wc -l)

nBases=$(cat $f | tr '\n' '\t' | sed 's/@channel/\n@channel/g'|cut -f 2|wc -c)

meanLength=$(python -c "print(round($nBases/float($nReads),1))")

alignedReads=$(cat $n\_reads.fq.true.read.stats|sed 's/ .*//g'|sort -u|wc -l)

meanAlignLen=$(cat $n\_reads.fq.true.read.stats|tr ' ' '\t'|cut -f 6,7|awk '{sum+=$2; sum-=$1; n+=1}END{print sum/n}')

meanIden=$(cat $n\_reads.fq.true.read.stats|tr ' ' '\t'|awk '{sum+=$4; n+=1}END{print sum/n}')

echo "$n $nReads $nBases $meanLength $alignedReads $meanAlignLen $meanIden"

done >> read.counts.tsv

**TAXONOMIC SPECIFICITY**

#!/bin/sh

#Count the number of bases aligned to the correct genus, species, and strain, as well as the incorrect genus

count_bases(){

fp=$1

term=$2

for read in $(egrep $term $fp | cut -d " " -f 1 | sort -u); do

egrep $term $fp | grep $read | cut -d " " -f 1,6,7 | awk '{print $1 "\t" $3-$2}'|sort -u | sort -nrk2 | head -1

done | awk '{sum+=$2}END{print sum}'

}

count_bases_absent(){

fp=$1

term=$2

for read in $(egrep -v $term $fp | cut -d " " -f 1 | sort -u); do

egrep -v $term $fp | grep $read | cut -d " " -f 1,6,7 | awk '{print $1 "\t" $3-$2}'|sort -u | sort -nrk2 | head -1

done | awk '{sum+=$2}END{print sum}'

}

count_reads(){

fp=$1

term=$2

egrep $term $fp | cut -d " " -f 1 | sort -u | wc -l

}

count_reads_absent(){

fp=$1

term=$2

egrep -v $term $fp | cut -d " " -f 1 | sort -u | wc -l

}

taxon(){

fp=$1

genus_reads=$(count_reads $fp $2)

genus_bases=$(count_bases $fp $2)

species_reads=$(count_reads $fp $3)

species_bases=$(count_bases $fp $3)

nongenus_reads=$(count_reads_absent $fp $2)

nongenus_bases=$(count_bases_absent $fp $2)

echo "$fp $genus_reads/$genus_bases $species_reads/$species_bases $nongenus_reads/$nongenus_bases"

}

echo "Dataset GenusReads/GenusBases SpeciesReads/SpeciesBases NonGenusReads/NonGenusBases" > taxonomic_specificity.tsv

taxon ecoli_reads.fq.read.stats 'Escherichia|Shigella' coli >> taxonomic_specificity.tsv

taxon MVA_reads.fq.read.stats 'Vaccinia|Cowpox' 'MVA|Cowpox' >> taxonomic_specificity.tsv

taxon lister_reads.fq.read.stats Vaccinia Lister >> taxonomic_specificity.tsv

taxon cowpox_reads.fq.read.stats Cowpox Cowpox >> taxonomic_specificity.tsv
